# Tissue Iron Distribution in Anemic Patients with End-Stage Kidney Disease: Results of a Pilot Study

**DOI:** 10.3390/jcm13123487

**Published:** 2024-06-14

**Authors:** Lukas Lanser, Michaela Plaikner, Josia Fauser, Verena Petzer, Sara Denicolò, David Haschka, Hannes Neuwirt, Kiril Stefanow, Michael Rudnicki, Christian Kremser, Benjamin Henninger, Guenter Weiss

**Affiliations:** 1 Department of Internal Medicine II, Medical University of Innsbruck, 6020 Innsbruck, Austria; 2 Department of Radiology, Medical University of Innsbruck, 6020 Innsbruck, Austria; 3 Department of Internal Medicine V, Medical University of Innsbruck, 6020 Innsbruck, Austria; 4 Department of Internal Medicine IV, Medical University of Innsbruck, 6020 Innsbruck, Austria

**Keywords:** anemia, chronic kidney disease, end-stage kidney disease, iron overload, magnet resonance imaging

## Abstract

**Background/Objectives**: Anemia is a frequent multifactorial co-morbidity in end-stage kidney disease (ESKD) associated with morbidity and poor QoL. Apart from insufficient erythropoietin formation, iron deficiency (ID) contributes to anemia development. Identifying patients in need of iron supplementation with current ID definitions is difficult since no good biomarker is available to detect actual iron needs. Therefore, new diagnostic tools to guide therapy are needed. **Methods**: We performed a prospective cohort study analyzing tissue iron content with MRI-based R2*-relaxometry in 20 anemic ESKD patients and linked it with iron biomarkers in comparison to 20 otherwise healthy individuals. **Results**: ESKD patients had significantly higher liver (90.1 s^−1^ vs. 36.1 s^−1^, *p* < 0.001) and spleen R2* values (119.8 s^−1^ vs. 19.3 s^−1^, *p* < 0.001) compared to otherwise healthy individuals, while their pancreas and heart R2* values did not significantly differ. Out of the 20 ESKD patients, 17 had elevated spleen and 12 had elevated liver R2* values. KDIGO guidelines (focusing on serum iron parameters) would recommend iron supplementation in seven patients with elevated spleen and four patients with elevated liver R2* values. **Conclusions**: These findings highlight that liver and especially spleen iron concentrations are significantly higher in ESKD patients compared to controls. Tissue iron overload diverged from classical iron parameters suggesting need of iron supplementation. Measurement of MRI-guided tissue iron distribution might help guide treatment of anemic ESKD patients.

## 1. Introduction

Anemia is a frequent comorbidity in patients with end-stage kidney disease (ESKD) and is associated with poor quality of life [1] and increased morbidity and mortality [2,3]. The prevalence of anemia increases with declining kidney function and reaches a prevalence of more than 50% in ESKD patients [4,5].

Several mechanisms are involved in the pathogenesis of this type of anemia [6]. Primarily, the reduced erythropoietin production as a consequence of kidney damage and dysfunction reduces erythropoietic bone marrow activity [7,8]. Reduced kidney blood flow in chronic kidney disease (CKD) alters oxygen delivery, with adaptation of the kidney tissue, subsequently consuming less oxygen with maintenance of a normal tissue oxygen gradient. Therefore, Epo production is not upregulated despite systemic hypoxia due to anemia [9]. In addition, inflammation inhibits the proliferation and differentiation of erythroid progenitor cells, raises their resistance to Epo and reduces the circulatory erythrocyte lifespan due to increased erythrophagocytosis by activated macrophages. In addition, the liver-derived iron hormone hepcidin was shown to inhibit erythroid progenitor proliferation and survival [10,11,12,13].

A further contributor to anemia in CKD is the reduced availability of iron, which is obligatory for an adequate erythropoietic response following stimulation by erythropoietin [14]. Moreover, iron is crucial for numerous biologic functions including cell respiration, energy production, and cell proliferation [15]. Actually, iron deficiency (ID) is associated with an adverse outcome in CKD even independently of anemia [16]. The origin of ID in CKD is multifactorial and caused by blood losses (blood left in the hemodialysis circuit, uremia-induced platelet dysfunction) [8] and reduced intestinal iron absorption together with inflammation-driven iron restriction in macrophages [17,18]. The latter is largely caused by increased circulating levels of hepcidin as a consequence of cytokine mediated induction of hepcidin but also reduced excretion of the peptide by the kidney [17,18,19]. Hepcidin binds to the iron exporter ferroportin, resulting in its degradation and the blockage of iron transfer from macrophages or enterocytes to the circulation [20]. Moreover, circulating cytokines, which have been found to be increased specifically in advanced or hemodialysis-dependent ESDR, induce the expression of transcellular iron uptake molecules, thereby enforcing macrophage iron storage, which is also reflected by increased ferritin levels [16,21].

Intravenous iron has been shown to reduce the needs of erythropoiesis-stimulating agents (ESAs) and blood transfusions, while increasing cognitive function and QoL in ESKD patients [22,23]. The PIVOTAL trial demonstrated that proactive intravenous iron administration in patients with ferritin < 700 mg/L and transferrin saturation (TfS) ≤40% not only decreases ESA use but also lowers the risk of all-cause death and cardiovascular events when compared to a reactive treatment strategy with low-dose intravenous iron administration in patients with ferritin < 200 mg/L and TfS < 20% [22]. Anemia correction with ESAs alone is associated with adverse outcomes [24]. Therefore, both the KDIGO and NICE guidelines recommend intravenous iron formulations for the treatment of anemia in ESKD patients, subsequently resulting in a change of practice patterns toward increased iron supplementation and reduced ESA use in the last several years [24,25].

Moreover, the response rates to intravenous iron therapy vary greatly, which may be traced back to the underlying type of ID. While it is suggestive that in subjects with true ID, even in the setting of chronic inflammatory diseases iron supplementation will be effective, this may not be the case in functional ID, as recently demonstrated in a pre-clinical model of inflammatory anemia where iron supplementation in the later setting resulted in accumulation of the metal in the liver and spleen without ameliorating the anemia [26].

As we lack good biomarkers to indicate true ID in the setting of inflammation [27], novel diagnostic tools are needed to guide therapy. Excess iron can cause radical formation and molecular damage and promote cell death and tissue injury, which is why it is important to avoid overtreatment of iron repleted or already overloaded patients [28]. A recent meta-analysis demonstrated a high risk of iron overload in patients with ESKD [29], which can cause intravascular oxidative stress and increase the risk of cardiovascular events and infections [27,30].

The aim of this study was (i) to evaluate tissue iron distribution in the spleen, liver, pancreas, and heart with MRI-based R2* relaxometry in anemic ESKD patients; (ii) to compare tissue iron distribution with otherwise healthy controls and a small group of patients with transfusion-related iron overload (TRIO); and (iii) to correlate tissue iron loading with biomarkers of iron metabolism and inflammation as well as premedication. A proper definition of ID that can be used for identifying patients with a need for iron supplementation is lacking since no good biomarker is available for detecting actual iron needs. Therefore, new diagnostic tools to guide iron therapy are warranted.

## 2. Materials and Methods

### 2.1. Study Population

We performed a prospective cohort pilot study of 20 patients with dialysis-dependent ESKD and anemia at the Nephrology Department of the Medical University of Innsbruck treated between May 2020–January 2022. Inclusion criteria were age > 18 years, hemoglobin < 130 g/L in men and <120 g/L in women and exclusion criterion general contraindications for MRI. The control group (already used in another analysis) consisted of otherwise healthy individuals without signs of inflammation (CRP < 0.5 mg/dL, n = 20), who were recruited at our outpatient department, together with a cohort of TRIO patients (ferritin > 500 µg/L) with hemato-oncologic disease classed as “stable disease” (n = 10). The control group of otherwise healthy individuals without signs of inflammation included 9 mildly anemic and 11 non-anemic patients.

Patients’ data were extracted from the local clinical information system and anonymized. This study conformed to the ethical principles outlined in the Declaration of Helsinki and was approved by the ethics committee of the Medical University of Innsbruck (ethical vote ID: UN5093, session number 325/4.14). All patients gave written informed consent to participate in this study. The data that support the findings of this study are available from the corresponding author upon reasonable request.

### 2.2. Laboratory Measurements and Classifications

Blood sampling was routinely performed at the time of imaging (±2 days) and analyzed with fully automated tests. Laboratory parameters were then extracted from the local clinical information system. Immunoturbidimetry tests were used for measuring ferritin, transferrin (Tf), soluble transferrin receptor (sTfR) and C-reactive protein (CRP) (Roche Diagnostics GmbH, Mannheim, Germany). The FerroZineTM method without deproteinization was used to measure serum iron concentrations (Roche Diagnostics GmbH, Mannheim, Germany). The TfS was calculated as iron/transferrin × 70.9 and the sTfR/ferritin index (sTfR-F) as sTfR/log (ferritin). An enzyme-linked immunosorbent assay was used to measure hepcidin (Siemens Healthcare Diagnostics Products GmbH, Marburg, Germany) and neopterin levels (IBL International GmbH, Hamburg, Germany), while flow cytometry was used to detect the blood count (Sysmex GmbH, Norderstedt, Germany). Finally, an electrochemiluminescence immunoassay was used to measure interleukin 6 (IL-6) and N-terminal prohormone of brain natriuretic peptide (NT-proBNP) levels (Roche Diagnostics GmbH, Mannheim, Germany).

We defined anemia according to the World Health Organization (WHO) definition as Hb < 130 g/L in men and Hb < 120 g/L in women and further classified it into severe (Hb < 80 g/L), moderate (Hb 80–109 g/L) and mild anemia (Hb 110–129 g/L in men, Hb 110–119 g/L in women) [31]. The KDIGO defines ID in ESKD patients as TfS ≤ 30% and ferritin ≤ 500 ng/mL [32].

### 2.3. Procedures of Magnetic Resonance Imaging

We referred study participants to the radiology department for MRI-based R2* relaxometry of the spleen, liver, pancreas, and T2* mapping of the heart. MRI of the upper abdominal organs (liver, spleen, and pancreas) was performed with a 1.5 T scanner (MAGNETOM AvantoFit, Siemens Healthineers, Erlangen, Germany) using an 18-element body matrix coil and 12–16 elements of the integrated 32-channel spine matrix coil. R2* values were acquired by means of a fat-saturated biopsy-calibrated [33] 2D multi-gradient echo (ME-GRE) sequence. Two transversal 10 mm thick single slices with 12 echoes were acquired at the hilar level of the liver. The sequence parameters were as follows: 0.99 ms initial TE, 1.41 ms delta TE, 16.5 ms max. TE, 200 ms TR, 20° flip angle, 128 × 128 matrix, 380 mm × 380 mm field of view, chemical shift selective fat saturation as provided by the manufacturer, and 16.8 s acquisition time. Images were taken in the supine position and at the end of expiration in one breath-hold per slice. The R2* maps were calculated by a blinded physicist, an expert in MRI post-processing. A plugin that was written specifically for ImageJ (Wayne Rasband, National Institutes of Health) was used for pixel-wise fitting with a mono-exponential truncation model [34]. The R2* maps were analyzed by a radiologist who carefully placed three approximately 8 mm in diameter “regions of interest (ROIs)” in the subcapsular liver parenchyma, positioning two in the right and one in the left lobe, two within the pancreas and one with about 13 mm in the center of the spleen. The liver surface, focal liver lesions and larger vessels as well as the artefacts were spared accordingly. For the liver and pancreas, the mean R2* values of the individual ROIs were used for further analysis. In addition, the proton density fraction (PDFF) was calculated by performing a commercial 3D multigradient-echo sequence (q-Dixon) with advanced in-line processing and automatic calculation of T1 weighted fat-saturated images and corresponding PDFF maps. The following sequence parameters were used: 64 slices, parallel imaging acceleration factor 4, acquisition time 18.5 s, 6 echoes, a 160 × 120 matrix, and FOV of 380 mm × 380 mm with a slice thickness of 3.5 mm. Tissue fat content of the liver, pancreas and spleen was determined from the ROIs manually co-registered to the R2* maps. To quantify the myocardial iron load, short-axis T2* maps were generated, and the largest possible ROI was placed within the mid-myocardial septum using the routine picture archiving and communication system (IMPACS, Agfa-Gevaert, Mortsel, Belgium). Cardiac R2* values were derived from the obtained T2* values by calculating the reciprocal value [35].

After defining the maximum organ length and height as well as the hilar thickness on the T1-weighted, fat-saturated q-Dixon images in the IMPACS system, the spleen volume was calculated using the following formula: spleen volume = 0.601 × (maximal length × vertical height × hilum thickness) + 18.889) [36].

Elevation of iron content was defined as R2* > 70 s^−1^ in the liver and as >50 s^−1^ in the spleen, pancreas, and heart according to the literature [35,37].

### 2.4. Statistical Analysis

We depicted parameters as n (%) or medians (25th, 75th percentile) since most parameters did not have a normal distribution as tested with Shapiro–Wilk. To test for significant differences between two or more unpaired groups we used the Mann-Whitney-U, Kruskal–Wallis or Pearson chi-square test. Correlations of the continuous variables were analyzed with Spearman-rank correlation analysis. Univariate and multivariate linear regression analysis was performed to analyze the predictive values of specific variables for the spleen and liver R2* values. Skewed variables were log-transformed by the natural logarithm for regression analysis. Statistical tests were two tailed and *p*-values < 0.05 were regarded as statistically significant. Statistical analysis was performed with SPSS Statistics Version 29 (IBM Corporation, Armonk, NY, USA).

## 3. Results

### 3.1. Patient Characteristics

We enrolled 20 ESKD patients with anemia of whom 16 were men and four women. The control group comprised 20 otherwise healthy individuals (ten men, ten women) and ten patients with TRIO (seven men, three women). The patients’ characteristics are depicted in Table 1. The R2* values in our control group were similar to healthy controls from recent studies [38,39,40]. The median time from. dialysis initiation until inclusion in this study was 18 months (2–41 months).

### 3.2. Tissue Iron Distribution Measured by MRI R2* Sequence

**Figure 1:**
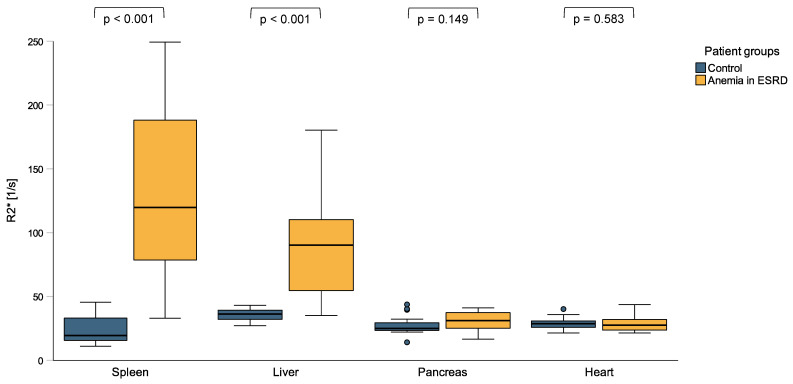
R2* values of spleen, liver, pancreas, and heart in patients with anemia in end-stage kidney disease (ESKD) and a control group of individuals without signs of inflammation. ESKD patients with anemia had significantly higher spleen and liver R2* values compared to otherwise healthy controls, while their pancreas and heart R2* values did not significantly differ.

**Figure 2:**
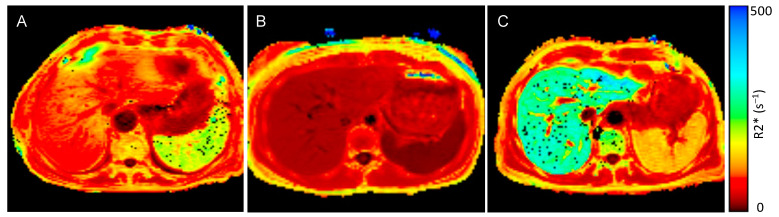
MRI R2* map of a patient with anemia and end-stage kidney disease (ESKD), control individual, and a patient with transfusion-related iron overload: (**A**) R2* maps of an anemic ESKD patient (spleen R2* value 189.7 s^−1^, liver R2* value 88.4 s^−1^), (**B**) R2* map of an otherwise healthy control individual without inflammation (spleen R2* value 15.3 s^−1^, liver R2* value 29.1 s^−1^) and (**C**) a patient with transfusion-related iron overload (spleen R2* value 126.0 s^−1^, liver R2* value 290.3 s^−1^)—dark red represents low R2* values and low iron concentrations, bright red and orange represents higher R2* values and higher iron concentrations, yellow and green represent the highest iron concentrations.

Patients with ESKD and anemia had significantly higher spleen (119.8 s^−1^ vs. 19.3 s^−1^, *p* < 0.001) and liver R2* values (90.1 s^−1^ vs. 36.1 s^−1^, *p* < 0.001) compared to healthy controls, while pancreas (31.0 s^−1^ vs. 24.9 s^−1^, *p* = 0.149) and heart R2* values (27.4 s^−1^ vs. 28.6 s^−1^, *p* = 0.583) did not significantly differ between the groups (Figure 1 and Figure 2). When applying the reference values used at our clinic [35], 85% of the ESKD patients (n = 17) had elevated spleen and 60% (n = 12) elevated liver R2* values, while their pancreas and heart iron R2* values were within the normal range.

The duration from the initiation of first hemodialysis was positively associated with spleen (rs = 0.618, *p* = 0.004) and liver R2* values (rs = 0.535, *p* = 0.015) but not with pancreas (rs = 0.233, *p* = −0.279) and heart R2* values (rs = 0.401, *p* = 0.080). We then analyzed the fat content of the spleen and liver by MRI-proton density fat fraction (MRI-PDFF), which accurately measures the fat fraction of tissue by correcting factors influencing the magnetic resonance signal intensity. We found that anemic ESKD patients had significantly higher spleen PDFF-values compared to controls (3.4% vs. 1.8%, *p* = 0.005), while there was also a trend for higher liver PDFF-values (2.3% vs. 1.7%, *p* = 0.086). Finally, the spleen volume was also significantly higher in anemic ESKD patients compared to controls (351.8 cm^3^ vs. 223.7 cm^3^, *p* = 0.001) (Table 1).

R2* values of liver (90.1 s^−1^ vs. 135.7 s^−1^, *p* = 0.169), spleen (119.8 s^−1^ vs. 79.7 s^−1^, *p* = 0.169), pancreas (31.0 s^−1^ vs. 30.8 s^−1^, *p* = 0.231) and heart (27.4 s^−1^ vs. 27.0 s^−1^, *p* = 0.650), and the spleen volume of anemic ESKD patients did not significantly differ when compared to a small cohort of patients with mild TRIO (a median of 10 RBC transfusions within the last year). Conversely, patients with TRIO had a significantly higher spleen volume compared to anemic ESKD patients (572.3 cm^3^ vs. 351.8 cm^3^, *p* = 0.001), while they tended to have lower spleen PDFF-values (1.5% vs. 3.4%, *p* = 0.055).

### 3.3. Peripheral Iron Measurements Do Not Reflect Tissue Iron Distribution Appropriately

Ten patients with ESDR had ID (TfS ≤ 30% and ferritin ≤ 500 ng/mL) and received iron supplementation as recommended in the KDIGO guidelines [32]. Although these patients had significantly lower spleen (110.4 s^−1^ vs. 188.1 s^−1^, *p* = 0.029) and liver R2* values (63.0 s^−1^ vs. 99.5 s^−1^, *p* = 0.043) compared to those without ID, three patients had elevated spleen and liver R2* values, four patients had only elevated spleen R2* values and one patient had an elevated liver R2* value. Interestingly, all 10 patients without ID (KDIGO guidelines) had elevated spleen R2* values and 8 patients also had elevated liver R2* values.

When comparing patients with ferritin < 200 ng/mL (absolute ID, n = 3) and ferritin 200–500 ng/dL together with a TfS < 30% (combined ID, n = 7) we found that patients with absolute ID had lower liver (42.7 s^−1^ vs. 81.4 s^−1^) and spleen R2* values (40.5 s^−1^ vs. 140.3 s^−1^); yet, the low number of patients not allow for appropriate statistical analysis. Only one patient with absolute ID had elevated spleen R2* values, while no one had elevated liver R2* values. In the ROC analysis, a ferritin of 340 ng/mL was best to discriminate between normal and elevated spleen R2* values (sensitivity 87.5%, specificity 100%, AUC 0.958 [95%CI 0.862–1.055]), while a ferritin of 490 ng/mL was best to discriminate between normal and elevated liver R2* values (sensitivity 58.3%, specificity 85.7%, AUC 0.738 [95% CI 0.497–0.979]). For TfS, the ROC analysis revealed a cut-off of 19.5% to discriminate between normal and elevated spleen R2* values (sensitivity 62.5%, specificity 66.7%, AUC 0.635 [95% CI 0.259–1.012]), while a cut-off of 15.0% was best to discriminate between normal and elevated liver R2* values (sensitivity 100%, specificity 28.6%, AUC 0.601 [95% CI 0.330–0.873]).

### 3.4. Tissue Iron Distribution, Serum Iron Homeostasis and Inflammatory Biomarkers

**Figure 3:**
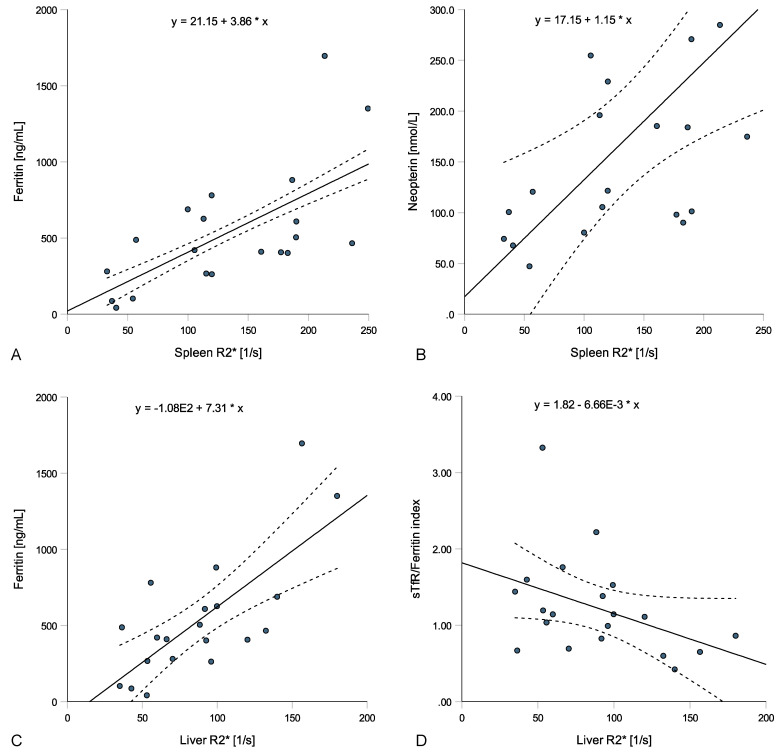
Relations of spleen and liver R2* values with laboratory parameters in patients with anemia in end-stage kidney disease (ESKD). Spleen R2* positively correlated with ferritin ((**A**); rs = 0.602, *p* = 0.005) and neopterin ((**B**); rs = 0.612, *p* = 0.004), while liver R2* positively correlated with ferritin ((**C**); rs = 0.624, *p* = 0.003) and negatively with the sTfR/ferritin index ((**D**); rs = −0.489, *p* = 0.029).

In anemic ESKD patients, spleen R2* values correlated with ferritin (rs = 0.602, *p* = 0.005, Figure 3A), transferrin (rs = −0.468, *p* = 0.038), hepcidin (rs = 0.481, *p* = 0.032) and neopterin concentrations (rs = 0.612, *p* = 0.004, Figure 3B) but not with CRP (rs = 0.144, *p* = 0.543) or IL-6 levels (rs = 0.139, *p* = 0.558), while liver R2* values correlated with ferritin (rs = 0.624, *p* = 0.003, Figure 3C), transferrin (rs = −0.567, *p* = 0.009) and the sTfR-F (rs = −0.489, *p* = 0.029, Figure 3D) and insignificantly with neopterin (rs = 0.436, *p* = 0.055) and hepcidin levels (rs = 0.395, *p* = 0.085). Pancreatic R2* values correlated only weakly with hepcidin levels (rs = −0.476, *p* = 0.034) and heart R2* values with transferrin levels (rs = −0.447, *p* = 0.048). Interestingly, the spleen volume correlated with CRP (rs = 0.537, *p* = 0.015), IL-6 (rs = 0.738, *p* < 0.001), Hb (rs = −0.624, *p* = 0.003) and Hk (rs = −0.534, *p* = 0.015), while no correlations with parameters of iron metabolism were found.

Since neopterin is excreted by the kidney, we also calculated the neopterin/creatinine ratio. The neopterin/creatinine ratio correlated with spleen (rs = 0.447, *p* = 0.048) and liver R2*-value* values (rs = 0.547, *p* = 0.012) but not with pancreas (rs = 0.005, *p* = 0.985) orand heart R2*-value* values (rs = 0.366, *p* = 0.112).

We performed linear regression analysis adjusted for age and stratified for sex, and we found that ferritin, Tf, hepcidin and neopterin, but not TfS, sTfR, sTfR-F, CRP or IL-6, predict spleen R2* values in anemic ESKD patients. When performing multivariate linear regression analysis stratified for sex and adjusted for age, ferritin, Tf, hepcidin and neopterin, only ferritin and neopterin were remaining remained as significant predictors offor spleen R2* values (Table 2).

In terms of liver R2* values, we found that ferritin, hepcidin, CRP and neopterin predicted liver R2* values in linear regression analysis adjusted for age and stratified for sex, while Tf, TfS, sTfR, sTfR-F and IL-6 did not. Again, when performing multivariate linear regression analysis stratified for sex and adjusted for age, ferritin, hepcidin, CRP and neopterin, only ferritin remained as a significant predictor offor liver R2* values *(*Table 2).

### 3.5. Iron Supplementation, ESA Therapy and Tissue Iron Distribution

Out of 20 ESKD patients, 14 patients received intravenous iron supplementation with FerMed^®^ and 19 patients received ESA therapy with Eporatio^®^ at the time of inclusion. The median iron dosage within the last year was 3550 mg (1500–4900 mg) and the median ESA dosage within the last year was 395,000 IU (296,000–705,000 IU). Patients with iron supplementation had higher liver R2* values compared to patients without iron supplementation, while spleen, pancreas, and heart R2* values as well as ferritin, TfS, hepcidin, sTfR, sTfR-F, and Hb did not differ (Table 3). However, the patient numbers were too small to perform appropriate statistical processing. Interestingly, no statistically significant correlation was found between iron dosage within the last year (in 19 patients with available dosing within the last year) and R2* values of liver (rs = 0.291, *p* = 0.226) and spleen (rs = 0.206, *p* = 0.397), while there was a negative correlation with hepcidin levels (rs = −0.471, *p* = 0.042) and parameters of iron metabolism.

ESA dose within the last year correlated with spleen R2* values (rs = 0.566, *p* = 0.012) but not with liver (rs = 0.292, *p* = 0.225), pancreas (rs = −0.136, *p* = 0.579) or heart R2* values (rs = −0.324, *p* = 0.175). ESA dose further correlated with Ret-Hb (rs = −0.476, *p* = 0.039), neopterin (rs = 0.494, *p* = 0.032) and the neopterin/creatinine ratio (rs = 0.497, *p* = 0.031), while again no correlation was found with parameters of iron metabolism or inflammation.

## 4. Discussion

Herein, we demonstrated that liver and especially spleen iron concentrations were significantly higher in anemic ESKD patients compared to healthy controls. According to the reference ranges, 85% had elevated spleen iron concentrations (SIC) and 60% elevated liver iron concentrations (LIC). SIC positively correlated with ferritin, transferrin, hepcidin, and neopterin, while LIC positively correlated with ferritin and transferrin. However, in multivariate linear regression analysis only ferritin remained a significant predictor for SIC and LIC. Of note, no increased iron load was detected in pancreas and heart. Iron can accumulate following continuous iron supplementation or due to recycled iron from damaged red blood cells (RBC) [41]. As a consequence, hemodialysis for a longer period of time would result in a higher risk for tissue iron accumulation. Actually, spleen and to a lesser extend liver R2* values were positively correlating with the duration since first hemodialysis.

Serum ferritin and TfS can be similar to those seen in patients with TRIO [42]. Accordingly, tissue iron concentrations did not significantly differ when compared to a small cohort of hemato-oncologic patients with mild TRIO.

Current guidelines recommend iron supplementation for treatment of anemia in ESKD based on cut-offs for serum iron biomarkers (TfS ≤ 30% and ferritin ≤ 500 ng/mL) [32]. Application of these guidelines to our cohort of ESKD patients identified 10 patients with a need for iron supplementation. However, 70% of these patients already had increased SIC and 40% already had increased LIC, suggesting an overtreatment with potentially more harmful effects including cell death, fibrosis, and carcinogenesis [43]. Thus, additional indicators of body iron status and tissue iron retention may help to better guide iron supplementation [44]. This could include MRI-based evaluation of body iron stores as our investigation indicated that a more restrictive ID definition in ESDR (e.g., ferritin < 350 ng/mL and TfS < 20%) may better identify patients who benefit from iron supplementation and are not at risk for iron overload. Also, other iron biomarkers including sTfR, hepcidin, erythroferrone or soluble hemojuvelin (sHJV) might be useful to better identify patients with a need for iron supplementation [45,46].

In the scientific literature, data on tissue iron distributions in ESKD patients are rare. Actually, a recent systemic review and meta-analysis could identify only seven studies that investigated tissue iron content by MRI-relaxometry in ESKD patients [29]. While six studies investigated LIC (heart iron concentrations were additionally measured in two studies), there was only one comparable study investigating the iron content of the liver, spleen, pancreas and heart in 21 patients with ESKD, whereas 100% had elevated SIC and 95% elevated LIC (when using the same reference ranges) [47]. However, the evaluated cohorts differed, especially concerning the Hb and ferritin levels, limiting the comparability of these results: Ghoti et al. included ESKD patients with intravenous iron therapy and ferritin >1000 ng/mL, while our study included ESKD patients with anemia with median ferritin levels of 444 ng/mL. This suggests that iron primarily accumulates in the spleen before being redistributed to the liver when iron overload progresses. Actually, erythrophagocytosis of damaged RBCs accumulating following chronic hemolysis during hemodialysis [48] primarily occurs in macrophages resident in the spleen [49]. The positive correlation of ESA dosage and SIC would support this finding since a shortened RBC lifespan was shown to be associated with ESA dose [50,51].

Nevertheless, transfused iron–carbohydrate complexes are taken up by macrophages and Kupffer cells [52], while studies using positron emission tomography demonstrated a slightly higher uptake in the liver [53]. Actually, patients who received iron supplementation within the last year had significantly higher liver but not spleen iron concentrations compared to patients without iron supplementation. These results suggest that intravenous iron might be primarily stored in the liver, while iron recycled from damaged RBCs might primarily accumulate in the spleen.

Neopterin, reflecting activation of monocytes and macrophages, and also hepcidin levels were correlated with spleen R2* values, while there was only a trend for correlation with liver R2* values, suggesting that inflammation-related iron restriction might primarily occur in the spleen. This is supported by animal models that demonstrated that macrophages withholding iron primarily accumulate in the spleen under inflammatory conditions [54,55], which could also be confirmed by a recent MRI investigation showing that patients with inflammatory anemia primarily accumulate iron in the spleen [56].

Limitations: We performed a pilot study that intended to investigate tissue iron distribution in a small cohort of anemic ESKD patients with preliminary results. Further studies with a higher number of patients are needed to verify these results with higher statistical power. The results of this study do not allow unrestricted generalization to the heterogenic group of ESKD patients. Although R2*-relaxometry is an established method for detecting iron concentrations in different tissues [40], there was no histologically verification by concomitant biopsies of the spleen until now. Previous studies used conversion formulas for liver iron concentrations to obtain spleen iron concentrations [39,57].

## 5. Conclusions

We demonstrated that liver and especially spleen iron concentrations were significantly higher in anemic ESKD patients compared to healthy controls, while elevated iron concentrations in pancreas and heart were not detected. A substantial proportion of ESKD patients with spleen and/or liver iron overload should receive iron supplementation when applying the current serum biomarker cut-offs according to the KDIGO guidelines. The duration since first hemodialysis was associated with both spleen and liver iron concentrations while the ESA dosage was associated with spleen iron concentrations and iron supplementation was associated with liver iron concentrations. Measurement of tissue iron distribution by MRI in addition to serum iron parameters might help guide iron supplementation therapy in some patients with ESKD in order to identify those who have already developed tissue iron overload, thus preventing overtreatment with potentially toxic effects.

## Figures and Tables

**Table 1:** Patients’ characteristics.

	Controls	ESKD Patients	Sig.
	n = 20	n = 20	
	Median (IQR) or %	Median (IQR) or %	*p*-Value
**Demographic and clinical characteristics**			
*Age [years]*	*30 (26–51)*	*63 (49–76)*	*<0.001*
*Sex [male]*	*50.0%*	*80.0%*	*0.047*
*BMI [kg/m^2^]*	*21.7 (20.3–22.5)*	*23.9 (21.9–27.4)*	*0.006*
**Comorbidities**			
*Arterial hypertension*	*5.0%*	*75.0%*	*<0.001*
*Hyperlipidemia*	*0.0%*	*35.0%*	*0.004*
Diabetes mellitus	10.0%	7.7%	0.945
*Coronary artery disease*	*10.0%*	*45.0%*	*0.013*
*Chronic heart failure*	*0.0%*	*20.0%*	*0.035*
*Acute infection*	*0.0%*	*50.0%*	*<0.001*
**Comedication**			
*Iron supplementation*	*0.0%*	*70.0%*	*<0.001*
Dosage within last year [IE]	*-*	*1650 (0–4750)*	*-*
*Erythropoiesis-stimulating agents*	*0.0%*	*95.0%*	*<0.001*
Dosage within last year [mg]	-	395,000 (296,000–705,000)	-
**Laboratory parameters**			
*Creatinine [mg/dL]*	*0.84 (0.68–0.95)*	*7.30 (5.47–9.35)*	*<0.001*
*Aspartate aminotransferase [U/L]*	*22 (18–28)*	*16 (14–20)*	*<0.001*
Alanine transaminase [U/L]	19 (15–23)	12 (9–22)	0.091
*Gamma-GT [U/L]*	*16 (14–22)*	*30 (16–58)*	*0.013*
*Alkaline phosphatase [U/L]*	*64 (51–79)*	*94 (75–148)*	*0.004*
Lactate dehydrogenase [U/L]	172 (153–192)	190 (163–223)	0.183
*NT-proBNP [ng/L]*	*49 (49–111)*	*5386 (2749–23,862)*	*<0.001*
*CRP [mg/dL]*	*0.10 (0.05–0.14)*	*0.54 (0.18–1.82)*	*<0.001*
*Interleukin 6 [ng/L]*	*1.4 (1.4–3.4)*	*6.3 (3.6–18.5)*	*<0.001*
*Neopterin [nmol/L]*	*6.9 (5.4–8.6)*	*121.1 (94.2–212.6)*	*<0.001*
**Blood count and iron parameters**			
Leucocytes [G/L]	6.5 (5.7–8.1)	5.8 (4.8–6.5)	0.091
*Lymphocytes [G/L]*	*2.18 (1.80–2.46)*	*1.21 (0.97–1.61)*	*<0.001*
*Erythrocytes [T/L]*	*4.46 (3.54–4.91)*	*3.28 (2.98–3.48)*	*<0.001*
*Hemoglobin [g/L]*	*129 (108–147)*	*100 (93–108)*	*<0.001*
*Hematocrit [L/L]*	*0.379 (0.324–0.415)*	*0.302 (0.274–0.320)*	*<0.001*
MCH [pg]	29.9 (28.0–31.2)	31.1 (30.1–32.0)	0.052
*MCV [fL]*	*85.2 (82.8–88.3)*	*91.9 (90.3–96.7)*	*<0.001*
MCHC [g/L]	344 (335–349)	331 (322–346)	0.183
*Red cell distribution width [%]*	*12.8 (12.2–14.4)*	*14.0 (13.3–15.2)*	*0.015*
*Platelets [G/L]*	*277 (243–360)*	*176 (151–239)*	*<0.001*
Reticulocytes [G/L]	57.0 (49.4–69.9)	49.6 (35.0–89.4)	0.461
Reticulocyte Hb [pg]	34 (31–35)	35 (31–37)	0.369
Serum iron [µmol/L]	10.5 (6.5–15.7)	9.4 (7.3–15.4)	0.989
*Ferritin [ng/mL]*	*32 (20–115)*	*444 (274–658)*	*<0.001*
*Transferrin [mg/dL]*	*282 (232–325)*	*178 (161–191)*	*<0.001*
*Transferrin saturation [%]*	*17 (9–24)*	*21 (17–30)*	*0.043*
sTfR [mg/L]	3.3 (2.8–4.2)	2.9 (2.2–3.4)	0.121
*sTfR/Ferritin index*	*1.90 (1.44–2.95)*	*1.13 (0.76–1.48)*	*<0.001*
*Hepcidin [µg/L]*	*3.1 (1.5–10.8)*	*82.0 (28.5–82.0)*	*<0.001*
Folic acid [µg/L]	7.5 (6.0–10.3)	6.4 (3.5–15.6)	0.425
Vitamin B12 [ng/L]	301 (216–427)	350 (275–534)	0.134
**MRI detections**			
Spleen R2* [s^−1^]	*19.3 (15.5–33.0)*	*119.8 (78.5–188.2)*	*<0.001*
*Spleen PDFF [%]*	*1.8 (1.1–2.2)*	*3.4 (1.7–5.7)*	*0.005*
*Spleen volume* [cm^3^]	*223.7 (180.1–292.8)*	*351.8 (239.6–410.3)*	*0.001*
Liver R2* [s^−1^*]*	*36.1 (31.9–39.1)*	*90.1 (54.5–110.0)*	*<0.001*
Liver PDFF [%]	1.7 (0.9–3.0)	2.3 (1.8–4.1)	0.086
Pancreas R2* [s^−1^]	195.3 (184.0–216.5)	258.3 (211.5–297.0)	0.149
Heart R2* [s^−1^]	24.9 (23.2–29.9)	31.0 (25.1–37.3)	0.583

Parameters of patients are listed as median (IQR) or n (%). Mann–Whitney test and the Pearson chi-square test were used for comparisons between ESKD patients and the control group. Sig. = significance; IQR = interquartile range; BMI = body mass index; NT-proBNP = N-terminal prohormone of brain natriuretic peptide; CRP = C-reactive protein; MCH = mean corpuscular hemoglobin; MCV = mean corpuscular volume; MCHC = mean corpuscular hemoglobin concentration; sTfR = soluble transferrin receptor; PDFF = proton density fat fraction.

**Table 2:** Linear regression analysis.

	Univariate Regression Analysis ^#^		Multivariate Regression Analysis ^#^	
	*β* (95% CI)	*p*-Value	*β* (95% CI)	*p*-Value
Prediction of spleen R2*				
*Ferritin*	*0.605 (0.352–0.857)*	*<0.001*	*0.580 (0.345–0.815)*	*<0.001*
*Transferrin*	*−1.816 (−3.240–−0.393)*	*0.016*	0.214 (−1.109–1.536)	0.733
Transferrin saturation	−0.121 (−0.551–0.308)	0.558		
*Hepcidin*	*0.413 (0.119–0.706)*	*0.009*	−0.223 (−0.630–0.184)	0.258
sTfR	0.249 (−0.673–1.169)	0.576		
sTfR-Ferritin index	−0.231 (−1.018–0.555)	0.542		
C-reactive protein	0.177 (−0.035–0.388)	0.096		
Interleukin 6	0.322 (−0.072–0.715)	0.102		
*Neopterin*	*0.644 (0.217–1.072)*	*0.006*	*0.530 (0.342–0.718)*	*<0.001*
Prediction of liver R2*				
*Ferritin*	*0.422 (0.234–0.609)*	*<0.001*	*0.633 (0.263–1.004)*	*0.003*
Transferrin saturation	−1.085 (−2.170–0.000)	0.050		
TfS	0.376 (0.071–0.824)	0.094		
*Hepcidin*	*0.236 (0.007–0.466)*	*0.044*	0.409 (−0.381–1.198)	0.283
sTfR	−0.198 (−0.854–0.456)	0.532		
sTfR-Ferritin index	−0.428 (−0.950–0.093)	0.101		
*C-reactive protein*	*0.177 (0.040–0.313)*	*0.014*	−0.223 (−0.563–0.116)	0.179
Interleukin 6	0.217 (−0.068–0.501)	0.126		
*Neopterin*	*0.420 (0.099–0.742)*	*0.014*	0.351 (−0.131–0.833)	0.140

Linear regression analysis for different laboratory markers in predicting spleen and liver R2*. Parameters were log-transformed by the natural logarithm since they did not follow a normal distribution. Parameters that were significant in univariate regression analysis were included in the multivariate regression analysis. ^#^ adjusted for age and stratified for sex. CI = confidence interval; sTfR = soluble transferrin receptor.

**Table 3:** Characteristics of patients with iron supplementation.

	No iron Supplementation	Iron Supplementation	Sig.
	n = 6	n = 14	
	Median (IQR) or %	Median (IQR) or %	*p*-Value
**Demographic and clinical characteristics**			
Age [years]	66 (63–73)	62 (48–76)	0.444
Sex [male]	83.3%	78.6%	0.807
BMI [kg/m^2^]	28.5 (22.2–32.6)	23.6 (21.8–25.0)	0.106
**Laboratory parameters**			
Creatinine [mg/dL]	8.32 (7.53–9.00)	6.08 (5.45–9.69)	0.109
Aspartate aminotransferase [U/L]	16 (14–18)	16 (13–22)	0.841
Alanine transaminase [U/L]	12 (9–32)	13 (9–21)	0.904
Gamma-GT [U/L]	47 (22–59)	28 (15–56)	0.353
Alkaline phoshatase [U/L]	76 (63–84)	125 (87–186)	0.051
Lactate dehydrogenase [U/L]	193 (161–229)	190 (165–216)	0.904
*NT-proBNP [ng/L]*	*23,862 (14,809–35,860)*	*2862 (2375–5597)*	*0.012*
CRP [mg/dL]	0.80 (0.05–2.60)	0.54 (0.19–1.40)	0.841
Interleukine 6 [ng/L]	13.9 (3.4–53.7)	5.6 (3.7–10.4)	0.397
Neopterin [nmol/L]	121.1 (105.5–185.3)	138.1 (90.2–229.2)	0.968
**Blood count and iron parameters**			
Leucocytes [G/L]	6.3 (5.6–6.5)	5.7 (4.6–6.3)	0.444
Lymphocytes [G/L]	1.24 (0.99–1.65)	1.21 (0.95–1.58)	0.841
Erythrocytes [T/L]	3.01 (2.95–3.33)	3.30 (2.98–3.55)	0.239
Hemoglobin [g/L]	9.6 (9.0–9.8)	10.4 (9.5–11.0)	0.051
Hematocrit [L/L]	0.284 (0.258–0.300)	0.313 (0.286–0.321)	0.076
MCH [pg]	30.2 (29.5–31.3)	31.3 (30.8–33.0)	0.207
MCV [fL]	91.7 (89.8–91.9)	92.8 (90.8–97.2)	0.494
MCHC [g/L]	328 (321–349)	331 (322–343)	0.841
Red cell distribution width [%]	14.3 (13.4–15.0)	13.9 (13.1–15.4)	0.841
Platelets [G/L]	168 (136–263)	184 (160–235)	0.904
Reticulocytes [G/L]	89.4 (35.3–97.0)	49.0 (34.7–65.0)	0.312
Reticulocyte Hb [pg]	36 (32–37)	34 (30–37)	0.659
Serum iron [µmol/L]	9.1 (8.3–12.5)	9.9 (7.2–17.6)	0.904
Ferritin [ng/mL]	416 (267–488)	486 (281–689)	0.602
Transferrin [mg/dL]	191 (124–214)	175 (162–185)	0.602
Transferrin saturation [%]	25 (20–29)	20 (17–30)	0.718
sTfR [mg/L]	3.0 (2.9–3.0)	2.8 (2.1–3.6)	0.779
sTfR/Ferritin index	1.17 (1.04–1.44)	1.05 (0.69–1.53)	0.602
Hepcidin [µg/L]	82.0 (29.5–82.0)	74.0 (27.5–82.0)	0.718
Folic acid [µg/L]	13.7 (4.8–21.0)	4.3 (3.3–8.7)	0.106
Vitamin B12 [ng/L]	298 (234–551)	408 (279–517)	0.397
**MRI detections**			
Spleen R2* [s^−1^]	110.4 (57.0–119.7)	180.0 (99.9–190.0)	0.207
Spleen PDFF [%]	3.0 (1.5–3.9)	3.6 (2.0–5.8)	0.779
Spleen volume [cm^3^]	379.1 (231.9–430.3)	330.7 (246.9–396.6)	0.659
Liver R2* [s^−1^]	*54.5 (36.4–59.8)*	*97.6 (88.4–132.4)*	*0.003*
Liver PDFF [%]	2.5 (1.6–2.9)	2.1 (1.9–5.3)	0.968
Pancreas R2* [s^−1^]	28.3 (24.2–36.1)	32.4 (27.0–37.6)	0.444
Heart R2* [s^−1^]	27.4 (25.6–27.8)	27.0 (23.3–33.3)	0.659

Parameters of patients are listed as median (IQR) or n (%). Mann–Whitney test and the Pearson chi-square test were used for comparisons between the two subgroups. However, the number of patients was rather low to perform appropriate statistical analyses. Sig. = significance; IQR = interquartile range; BMI = body mass index; NT-proBNP = N-terminal prohormone of brain natriuretic peptide; CRP = C-reactive protein; MCH = mean corpuscular hemoglobin; MCV = mean corpuscular volume; MCHC = mean corpuscular hemoglobin concentration; sTfR = soluble transferrin receptor; PDFF = proton density fat fraction.

## Data Availability

The data that support the findings of this study are available from the corresponding author upon reasonable request.
